# Primary hypertrophic osteoarthropathy type 2 presenting as an acromegaly mimic with enteropathy

**DOI:** 10.1210/jcemcr/luag342

**Published:** 2026-11-06

**Authors:** Sandeep Kumar, Samir Jung Khatri, T Sreevatsa, Amit Kumar, S K Singh, Suman Kumar

**Affiliations:** Department of Internal Medicine, Armed Forces Medical College, Pune 411040, India; Department of Medicine, Armed Forces Medical College, Pune 411040, India; Department of Medicine, Command Hospital, Southern Command, Pune 411040, India; Department of Medicine, Command Hospital, Southern Command, Pune 411040, India; Department of Internal Medicine, Armed Forces Medical College, Pune 411040, India; Department of Internal Medicine, Armed Forces Medical College, Pune 411040, India

**Keywords:** acromegaly, primary hypertrophic osteoarthropathy, pachydermoperiostosis, *SLCO2A1*, chronic enteropathy associated with SLCO2A1, prostaglandin E2

## Abstract

Primary hypertrophic osteoarthropathy (PHO) is a rare genetic disorder characterized by digital clubbing, pachydermia, and periostosis that frequently mimics acromegaly. Autosomal recessive pathogenic variants in *SLCO2A1* cause PHO type 2 (PHOAR2) and are uniquely associated with chronic enteropathy associated with *SLCO2A1* (CEAS). We report a 39-year-old male evaluated for acromegaly due to progressive coarse facies, cutis verticis gyrata, and profound limb clubbing. Endocrine workup demonstrated a normal growth hormone (GH) suppression test (GH nadir 0.76 ng/mL [SI: 0.76 µg/L]; reference range, <1 ng/mL [SI: <1 ug/L]) and a low-normal insulin-like growth factor 1 of 78 ng/mL (SI: 10.2 nmol/L) (reference range, 80-220 ng/mL [SI: 10.5-28.8 nmol/L]), attributable to nutritional suppression from chronic enteropathy, definitively excluding acromegaly. Severe microcytic anemia (hemoglobin 5.9 g/dL [SI: 59 g/L]; reference range, 13.0-17.0 g/dL [SI: 130-170 g/L]) resulted from gastrointestinal bleeding. Deliberate terminal ileoscopy identified an ulcerated ileocecal valve confirming CEAS. Radiography showed extensive periostosis and a normal sella turcica. Next-generation sequencing identified a homozygous *SLCO2A1* pathogenic variant (c.764G>A, p.Gly255Glu), confirming PHOAR2-CEAS syndrome. Etoricoxib (120 mg daily) provided partial symptomatic relief. This case illustrates that digital clubbing in a patient with acromegaloid features mandates *SLCO2A1* genetic evaluation, avoiding unnecessary pituitary investigation.

## Introduction

Primary hypertrophic osteoarthropathy (PHO), historically known as pachydermoperiostosis, is a rare hereditary multisystem disorder defined by the triad of pachydermia (skin thickening), digital clubbing, and periostosis (subperiosteal bone formation) [1]. Because soft-tissue hypertrophy and skeletal proliferation prominently involve the face and acral extremities, PHO frequently masquerades as acromegaly, leading to extensive and unwarranted endocrine evaluations [2].

The pathophysiology of PHO stems from elevated prostaglandin E2 (PGE2) levels resulting from impaired degradation or membrane transport [3]. Primary hypertrophic osteoarthropathy is genetically heterogeneous. Pathogenic variants in *HPGD* (encoding 15-hydroxyprostaglandin dehydrogenase [15-PGDH]) cause PHO type 1 (PHOAR1), whereas pathogenic variants in *SLCO2A1* (encoding the prostaglandin transporter OATP2A1) cause PHO type 2 (PHOAR2). OATP2A1 mediates cellular PGE2 uptake; loss-of-function pathogenic variants impair this clearance, allowing unchecked PGE2 accumulation that drives osteoblastic proliferation, dermal fibroblast activation, and vascular remodeling [3, 4].

*SLCO2A1* is widely expressed in vascular endothelium and the gastrointestinal tract. Unlike *HPGD* pathogenic variants, *SLCO2A1* defects uniquely predispose patients to chronic enteropathy associated with *SLCO2A1* (CEAS), characterized by intractable small bowel and ileal ulcerations causing chronic occult blood loss and severe anemia [5]. Critically, CEAS is clinically and endoscopically distinct from inflammatory bowel disease (IBD), including Crohn disease, despite sharing features, such as ileal ulceration; unlike IBD, CEAS does not respond to standard immunosuppressive regimens, and molecular genetic diagnosis via *SLCO2A1* sequencing is required for definitive distinction [6]. We report a 39-year-old male presenting with an acromegaloid phenotype and severe gastrointestinal bleeding, diagnosed via clinical clues and molecular genetic diagnosis.

## Case presentation

A 39-year-old male was referred to the endocrinology clinic for evaluation of progressive acromegaloid features evolving over a 15-year period. His height was 168 cm (66.1 inches), weight 74 kg (162.8 lb), body mass index (BMI) 26.2 kg/m^2^, blood pressure 122/78 mmHg, heart rate 78 beats per minute, and respiratory rate 16 per minute. He noted gradual coarsening of facial skin, widening of the hands and feet, and excessive scalp folding. Physical examination revealed striking cutis verticis gyrata, a cerebriform, deeply furrowed thickening of the scalp, and coarse facial features (Fig. 1A and C). Prominent digital clubbing was present in all digits of both upper and lower extremities, with bilateral diffuse pachydermia of the hands and feet (Fig. 1B and D). Family history was notable for a sister who also exhibited prominent swelling of the distal fingers, a phenotypic finding that is inconsistent with acromegaly—not characterized by familial digital clubbing—and that strongly suggested a hereditary periostosing syndrome segregating in an autosomal recessive pattern.

**Figure 1:**
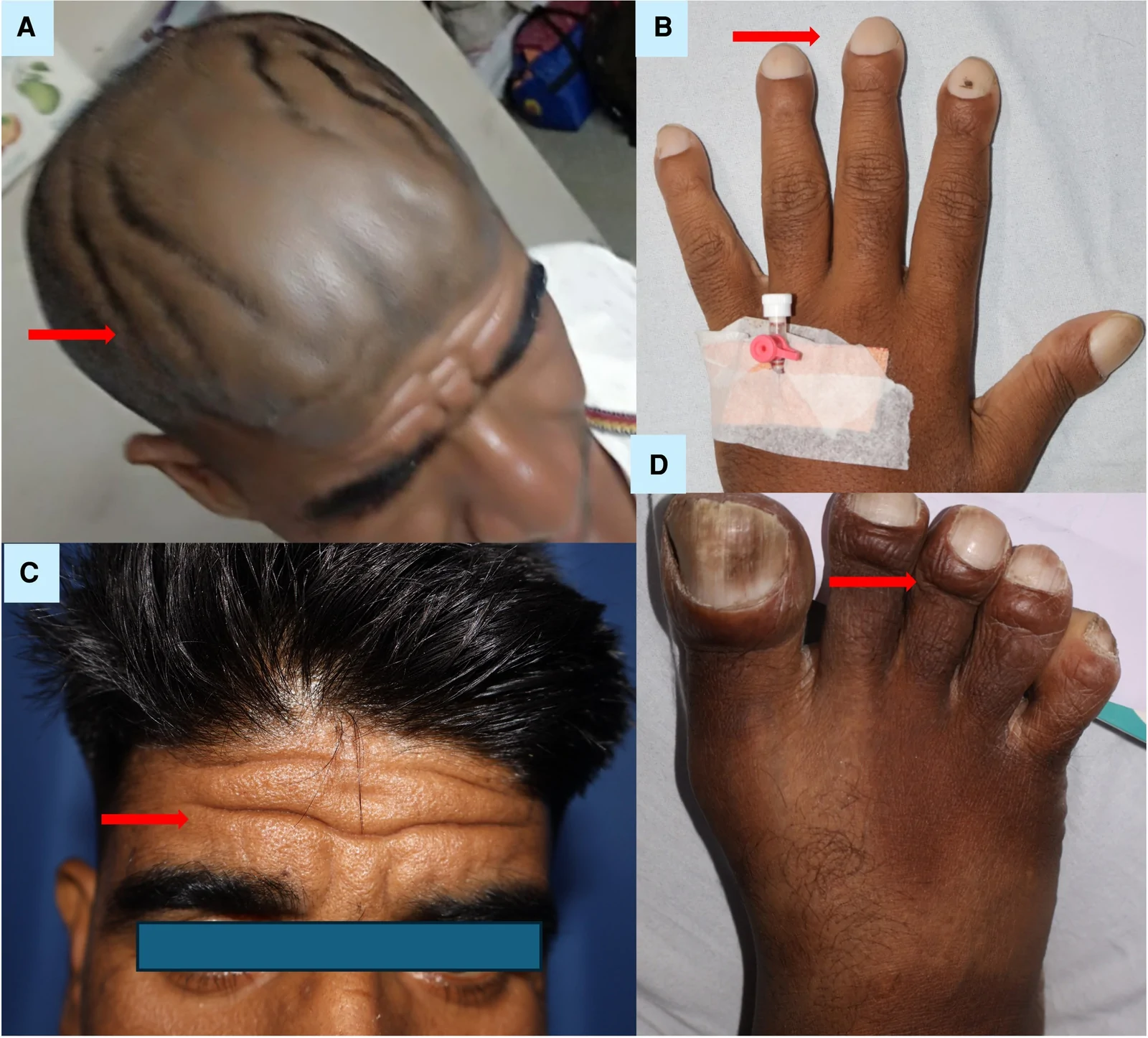
Clinical phenotype of the 39-year-old male patient presenting with an acromegaly mimic. (A) Lateral view of the scalp demonstrating severe cutis verticis gyrata (deep, cerebriform scalp furrows) (arrowed). (B) Dorsal view of the hand showing pronounced digital clubbing (arrowed) with a peripheral IV cannula in situ. (C) Frontal facial photograph showing marked coarsening of the facial skin and deep forehead furrows (arrowed); patient identity obscured. (D) Dorsal view of the feet demonstrating bilateral, symmetrical digital clubbing of the toes (arrowed) and diffuse pachydermia of the forefoot.

## Diagnostic assessment

To investigate suspected acromegaly, a comprehensive baseline endocrine evaluation was performed. Serum insulin-like growth factor 1 (IGF-1) was 78 ng/mL (SI: 10.2 nmol/L) (reference range, 80-220 ng/mL [SI: 10.5-28.8 nmol/L]), and free thyroxine (FT4) was 1.2 ng/dL (SI: 15.4 pmol/L) (reference range, 0.8-1.8 ng/dL [SI: 10.3-23.2 pmol/L]) (Table 1). A 75 g oral glucose tolerance test (OGTT) demonstrated complete growth hormone (GH) suppression with a GH nadir of 0.76 ng/mL (SI: 0.76 µg/L), definitively excluding somatotroph hypersecretion. The low-normal IGF-1 concentration is attributable to nutritional suppression in the context of chronic gastrointestinal blood loss, hypoalbuminemia (albumin 3.1 g/dL [SI: 31 g/L]; reference range, 3.5-5.0 g/dL [SI: 35-50 g/L]), and systemic inflammation (C-reactive protein [CRP] 18 mg/L [SI: 171 nmol/L]; reference range, <5 mg/L [SI: <47.6 nmol/L])—states in which IGF-1 falls independently of GH secretory status. A lateral skull radiograph revealed a normal sella turcica (Fig. 2C), providing anatomical corroboration against a pituitary macroadenoma.

**Figure 2:**
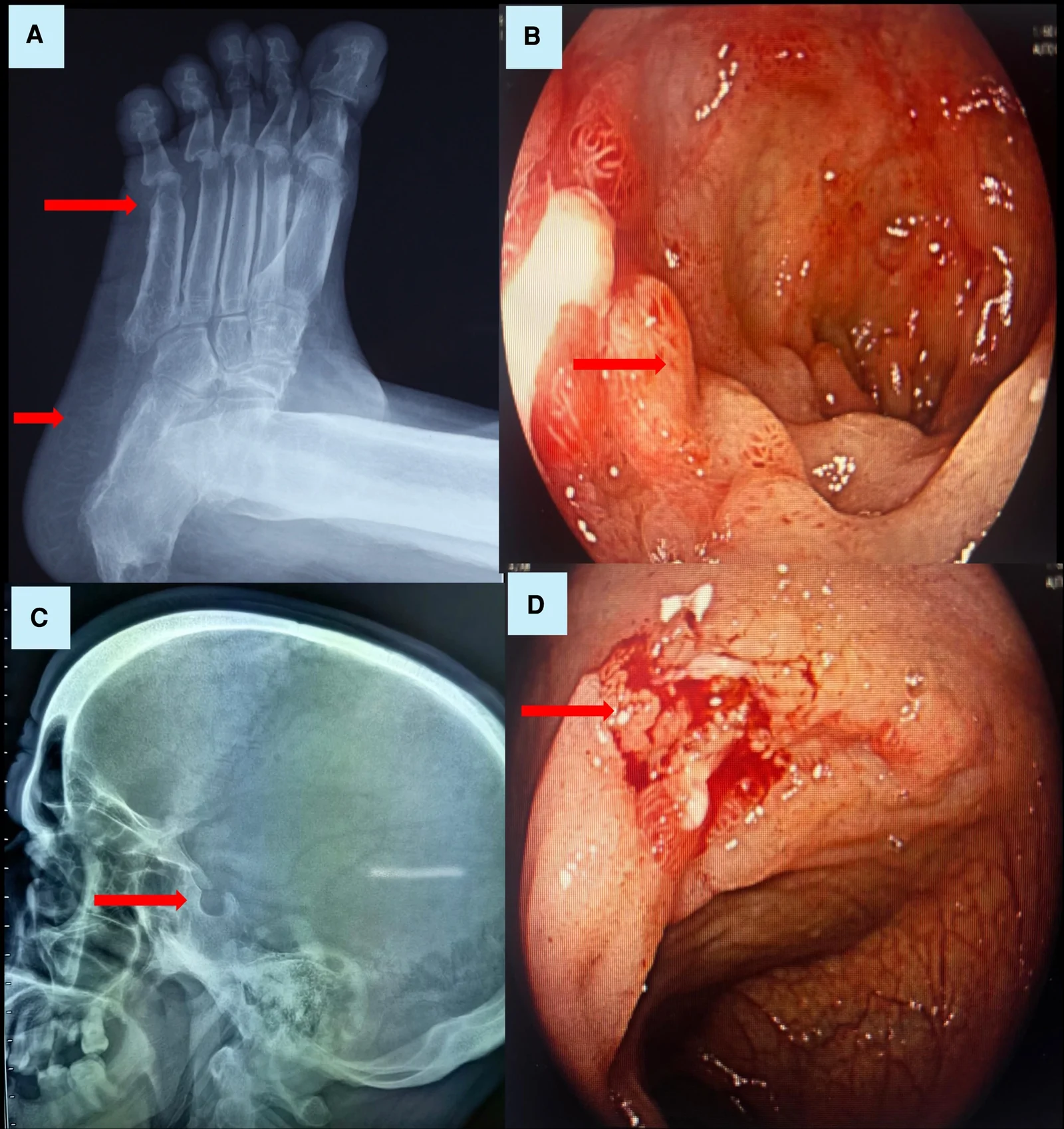
Radiographic and endoscopic findings. (A) Lateral radiograph of the right foot demonstrating pronounced periostosis with cortical thickening and subperiosteal new bone formation along the metatarsal diaphyses (arrowed). (B) Colonoscopic view showing the ileocecal region with mild mucosal erythema and a narrowed ileocecal valve. (C) Lateral skull radiograph revealing a normal sella turcica (arrowed), excluding a pituitary macroadenoma and providing anatomical corroboration against somatotroph hypersecretion. (D) Colonoscopic view of the ulcerated ileocecal valve with active mucosal hemorrhage (arrowed), confirming terminal ileitis characteristic of CEAS.

**Table 1:** Key laboratory and endocrine investigations

Parameter	Patient result, conventional (SI units below)	Reference range, conventional (SI below)	Clinical interpretation
**Hematology**			
Hemoglobin (Hb)	5.9 g/dL ↓ *SI: 59 g/L* ↓	13.0 to 17.0 g/dL *SI: 130 to 170 g/L*	Severe microcytic hypochromic anemia (CEAS bleeding)
Mean corpuscular volume (MCV)	70.7 fL ↓ *SI: 70.7* *fL* ↓	80.0 to 100.0 fL *SI: 80.0 to 100.0* *fL*	Consistent with chronic iron deficiency
White blood cell count (WBC)	6.2 × 10^3^/µL *SI: 6.2* *×* *10^9^/L*	4.0 to 11.0 × 10^3^/µL *SI: 4.0 to 11.0* *×* *10^9^/L*	Normal (excludes myelosuppression)
Platelet count	280 × 10^3^/µL *SI: 280* *×* *10^9^/L*	150 to 400 × 10^3^/µL *SI: 150 to 400* *×* *10^9^/L*	Normal (myelofibrosis surveillance—see footnote)
C-reactive protein (CRP)	18 mg/L ↑ *SI: 171 nmol/L* ↑	<5 mg/L *SI: <47.6 nmol/L*	Elevated—chronic systemic inflammation
Serum ferritin	4 ng/mL ↓ *SI: 4 *µ*g/L* ↓	24 to 336 ng/mL *SI: 24 to 336 *µ*g/L*	Severely depleted—iron-deficiency anemia
Serum albumin	3.1 g/dL ↓ *SI: 31 g/L* ↓	3.5 to 5.0 g/dL *SI: 35 to 50 g/L*	Mild hypoalbuminemia—protein-caloric malnutrition
**Endocrinology and biochemistry**			
Insulin-like growth factor 1 (IGF-1)	78 ng/mL *SI: 10.2 nmol/L*	80 to 220 ng/mL *SI: 10.5 to 28.8 nmol/L*	Low-normal; nutritional suppression (see text)
Free thyroxine (FT4)	1.2 ng/dL *SI: 15.4 pmol/L*	0.8 to 1.8 ng/dL *SI: 10.3 to 23.2 pmol/L*	Euthyroid
Thyroid-stimulating hormone (TSH)	2.1 mIU/L *SI: 2.1 mIU/L*	0.4 to 4.0 mIU/L *SI: 0.4 to 4.0 mIU/L*	Normal
**75 g oral glucose tolerance test—gh suppression**			
GH at 0 minutes (basal)	1.40 ng/mL *SI: 1.40 *µ*g/L*	N/A (basal) *N/A (basal)*	Basal (preload)
GH at 30 minutes	0.82 ng/mL *SI: 0.82 *µ*g/L*	<1 ng/mL *SI: <1 *µ*g/L*	Normal suppression
GH at 60 minutes	0.88 ng/mL *SI: 0.88 *µ*g/L*	<1 ng/mL *SI: <1 *µ*g/L*	Normal suppression
GH at 90 minutes	0.95 ng/mL *SI: 0.95 *µ*g/L*	<1 ng/mL *SI: <1 *µ*g/L*	Normal suppression
GH at 120 minutes	0.76 ng/mL *SI: 0.76 *µ*g/L*	<1 ng/mL *SI: <1 *µ*g/L*	Normal suppression

SI units shown in italics below conventional units. ↓ = below reference range; ↑ = above reference range. Conversion factors: Hb g/dL × 10 = g/L; IGF-1 ng/mL × 0.131 = nmol/L; FT4 ng/dL × 12.87 = pmol/L; CRP mg/L × 9.524 = nmol/L; albumin g/dL × 10 = g/L; GH ng/mL = µg/L (numerically equivalent); WBC/platelet 10^3^/µL = 10^9^/L. WBC and platelet count: Normal values at baseline do not exclude future development of myelofibrosis, a recognized complication of germline *SLCO2A1* pathogenic variants; serial monitoring is warranted [7].

Abbreviations: CEAS, chronic enteropathy associated with *SLCO2A1*; CRP, C-reactive protein; FT4, free thyroxine; GH, growth hormone; IGF-1, insulin-like growth factor 1; MCV, mean corpuscular volume; TSH, thyroid-stimulating hormone; WBC, white blood cell count.

Concurrently, the patient reported hematochezia and generalized fatigue. Laboratory investigation revealed severe microcytic hypochromic anemia: hemoglobin 5.9 g/dL (SI: 59 g/L) (reference range, 13.0-17.0 g/dL [SI: 130-170 g/L]) and mean corpuscular volume (MCV) 70.7 fL (SI: 70.7 fL) (reference range, 80.0-100.0 fL), with a serum ferritin of 4 ng/mL (SI: 4 µg/L) (reference range, 24-336 ng/mL [SI: 24-336 µg/L]), indicative of chronic iron deficiency from gastrointestinal bleeding (Table 1). Although an initial outpatient colonoscopy was interpreted as unremarkable, a subsequent colonoscopy with deliberate intubation of the terminal ileum revealed a contracted cecum with a narrowed, ulcerated ileocecal valve (Fig. 2B and D), confirming chronic terminal ileitis—a hallmark of CEAS. Skeletal radiography demonstrated widespread periostosis characterized by cortical thickening and subperiosteal new bone formation along the diaphyses of the metatarsals and phalanges (Fig. 2A).

Given the constellation of pachydermoperiostosis, an acromegaloid phenotype, and chronic bleeding enteropathy, next generation sequencing (NGS) was performed. This identified a homozygous pathogenic missense variant in *SLCO2A1* (c.764G>A, p.Gly255Glu), confirming the diagnosis of PHOAR2-CEAS syndrome [5]. Homozygosity was determined by a variant allele frequency of approximately 100% at this locus on whole-exome sequencing (mean depth > 50×), with no alternate allele detected. The variant c.764G>A (p.Gly255Glu) is located within the fifth transmembrane domain (TM5) of OATP2A1, a region critical for PGE2 binding and transport. Substitution of glycine (nonpolar) with glutamic acid (negatively charged) at position 255 disrupts the hydrophobic transmembrane core, abolishing transporter function. This variant is cataloged in ClinVar (Variation ID: 450498) as pathogenic, consistent with American College of Medical Genetics and Genomics (ACMG) and Association for Molecular Pathology (AMP) classification criteria, and is conserved across all mammalian SLCO2A1 orthologs.

## Treatment

The patient received blood transfusion followed by oral iron supplementation for severe iron-deficiency anemia. Cyclooxygenase-2 (COX-2) inhibitor therapy was initiated with etoricoxib (120 mg daily), which yielded partial relief of bilateral arthralgia of the knees and ankles and diffuse soft-tissue tightness (pachydermia-associated discomfort). Hematochezia ceased within 4 weeks of treatment initiation, consistent with PGE2-mediated mucosal healing. Naproxen was added on an as-needed basis for breakthrough pain. The therapeutic rationale for COX-2 inhibition is reduction of upstream PGE2 synthesis, thereby attenuating both musculoskeletal and gastrointestinal manifestations of *SLCO2A1* deficiency [3]. Long-term management options discussed with the patient include bisphosphonates (zoledronic acid), which have been reported to reduce bone pain and attenuate periostosis in PHO [8], and oral retinoids for management of progressive pachydermia. Novel targeted therapies, including prostaglandin E receptor (EP4) antagonists, are under clinical investigation. Bisphosphonate therapy will be considered should arthralgia remain refractory to current COX-2 inhibitor therapy.

## Outcome and follow-up

Following molecular confirmation, genetic counseling was provided regarding the autosomal recessive inheritance of the *SLCO2A1* pathogenic variant, and *SLCO2A1* sequencing was recommended for the patient's similarly affected sister; formal phenotypic assessment and results will be reported in a future communication. The patient remains under close multidisciplinary surveillance involving gastroenterology and endocrinology. Endocrinology follow-up is maintained to (1) monitor bone metabolism given the profound periostosis and PGE2-mediated osteoblastic hyperactivity, with periodic assessment of bone mineral density by dual-energy X-ray absorptiometry (DEXA); (2) manage nutritional consequences of chronic enteropathy, including fat-soluble vitamin deficiencies; and (3) coordinate multidisciplinary and clinical genetics care. Periodic capsule endoscopy and iron studies are planned to monitor enteropathy progression. Measurement of bone turnover markers (procollagen type 1 N-terminal propeptide [P1NP], bone-specific alkaline phosphatase [BAP], and urinary N-telopeptide [NTX]) and urinary prostaglandin E metabolites is planned at the next visit to establish a biochemical baseline for monitoring treatment response.

## Discussion

This case highlights a classic yet often overlooked diagnostic challenge: an endocrine mimic in which multisystem cutaneous and skeletal alterations lead to unnecessary pituitary evaluations. The patient's acromegaloid phenotype is visually striking but ultimately deceptive. The key distinguishing feature, evident from the outset, was digital clubbing.

Acromegaly causes broad, spade-like hands and soft-tissue thickening driven by IGF-1-mediated growth but does not cause true clubbing with periostitis. Periostosis, the hallmark radiological feature of PHO, arises from PGE2-stimulated osteoblastic activity and is entirely absent in somatotroph adenoma-induced acromegaly. The presence of profound digital clubbing, cutis verticis gyrata, and periosteal new bone formation with a normal GH suppression test and low-normal IGF-1 should redirect the clinician toward hereditary skeletal-skin syndromes rather than pituitary imaging [8, 9].

The family history in this case carries significant diagnostic weight. The patient's sister manifests prominent swelling of the distal fingers, a phenotype consistent with *SLCO2A1* haploinsufficiency or homozygous pathogenic variants. Acromegaly is caused by a sporadic or, rarely, multiple endocrine neoplasia 1 (MEN1)-associated pituitary somatotroph adenoma and does not co-segregate as symmetrical clubbing in siblings. The co-occurrence of acromegaloid features and digital clubbing in 2 siblings is highly suggestive of a hereditary periostosing syndrome and must prompt *SLCO2A1* or *HPGD* genetic evaluation.

The low-normal IGF-1 (78 ng/mL [SI: 10.2 nmol/L]) in this patient merits specific discussion. Insulin-like growth factor 1 is a nutritional biomarker that is suppressed by undernutrition, hypoalbuminemia, hepatic dysfunction, and systemic inflammation, independently of GH secretory status. In this patient, severe iron-deficiency anemia, hypoalbuminemia, elevated CRP, and chronic protein-caloric malnutrition from CEAS provide a compelling nutritional basis for IGF-1 suppression. This finding reinforces that IGF-1 must not be interpreted in isolation; a dynamic GH suppression test, which was definitively normal (GH nadir 0.76 ng/mL [SI: 0.76 µg/L]—well below the diagnostic threshold of <1 µg/L), is the gold-standard test for acromegaly.

The molecular basis of PHOAR2 involves loss-of-function pathogenic variants in *SLCO2A1*, which disrupt OATP2A1-mediated cellular uptake and intracellular degradation of PGE2. Unchecked PGE2 accumulation stimulates osteoblast proliferation via cyclic AMP-dependent pathways and activates dermal fibroblasts, driving the periostosis and pachydermia that define the syndrome [3]. The autosomal recessive pattern explains the consanguinity or family clustering frequently observed.

The coexistence of CEAS provides the molecular fingerprint distinguishing PHOAR2 from PHOAR1. Because OATP2A1 is highly expressed in the gut vascular endothelium and mucosal epithelium, its deficiency allows local PGE2 excess, causing multiple shallow ulcers predominantly in the small bowel and terminal ileum. These lesions evade standard colonoscopy unless the terminal ileum is deliberately intubated—a step initially omitted in this patient [7]. Chronic enteropathy associated with *SLCO2A1* is frequently and harmfully misdiagnosed as Crohn disease; however, standard immunosuppressive therapies fail in CEAS since the primary pathology is prostaglandin mediated rather than immune mediated [6]. Recognition prevents iatrogenic harm.

Normal white blood cell count (WBC) and platelet count in this patient are clinically noteworthy. Germline *SLCO2A1* pathogenic variants predispose to myelofibrosis—a potentially life-threatening hematological complication—requiring serial peripheral blood count surveillance. Normal platelet count and WBC at presentation exclude overt myelofibrosis but warrant continued monitoring.

An important methodological limitation of this report is the absence of bone turnover markers (BAP, P1NP, osteocalcin, tartrate-resistant acid phosphatase 5b [TRAP5b], deoxypyridinoline [DPD], and amino-terminal cross-linking telopeptide of bone collagen [NTX]) and urinary PGE2 metabolites at baseline. These markers would have quantified disease activity and established a treatment monitoring framework. Their measurement is planned and will be reported prospectively.

## Learning points

- Primary hypertrophic osteoarthropathy featuring cutis verticis gyrata and pachydermia presents a striking visual mimic of acromegaly and may trigger expensive, unnecessary pituitary investigations if the clinician fails to recognize the cardinal distinguishing sign of true digital clubbing.
- True digital clubbing and periostosis are consistently absent in classical somatotroph adenoma-induced acromegaly; their unambiguous presence, particularly in the context of a positive family history of similar features in a sibling, immediately points toward a hereditary periostosing syndrome and mandates genetic evaluation rather than pituitary imaging.
- A normal growth hormone suppression test and age-sex-adjusted IGF-1 concentration make acromegaly a highly unlikely diagnosis and, in the appropriate clinical context, should prompt next-generation sequencing for *SLCO2A1* pathogenic variants; a low-normal IGF-1 in a patient with chronic malnutrition and systemic inflammation should be interpreted with caution.
- Autosomal recessive pathogenic variants in *SLCO2A1* uniquely link pachydermoperiostosis with CEAS, which manifests as refractory iron-deficiency anemia from chronic small bowel ulceration and must be distinguished from Crohn disease, as standard immunosuppressive therapy is ineffective and may cause harm.
- Chronic enteropathy associated with *SLCO2A1* causes small bowel and ileal ulcers that evade routine colonoscopy unless deliberate terminal ileoscopy is performed; furthermore, all patients with *SLCO2A1* pathogenic variants require long-term surveillance for myelofibrosis, and management with COX-2 inhibitors targets upstream PGE2 synthesis to attenuate both the rheumatologic and gastrointestinal manifestations of the disease.

## Data Availability

The *SLCO2A1* variant c.764G>A (p.Gly255Glu) is cataloged in ClinVar (Variation ID: 450498). All other data generated or analyzed during this study are included in this published article.
